# Ethanol Intoxication Impairs Respiratory Function and Bacterial Clearance and Is Associated With Neutrophil Accumulation in the Lung After *Streptococcus pneumoniae* Infection

**DOI:** 10.3389/fimmu.2022.884719

**Published:** 2022-05-04

**Authors:** Holly J. Hulsebus, Kevin M. Najarro, Rachel H. McMahan, Devin M. Boe, David J. Orlicky, Elizabeth J. Kovacs

**Affiliations:** ^1^Department of Surgery, Division of GI, Trauma and Endocrine Surgery, University of Colorado Anschutz Medical Campus, Aurora, CO, United States; ^2^Immunology Graduate Program, University of Colorado Anschutz Medical Campus, Aurora, CO, United States; ^3^Department of Pathology, University of Colorado Anschutz Medical Campus, Aurora, CO, United States

**Keywords:** alcohol, inflammation, innate immunity, lung function, macrophage, leukocyte

## Abstract

Alcohol consumption is commonplace in the United States and its prevalence has increased in recent years. Excessive alcohol use is linked to an increased risk of infections including pneumococcal pneumonia, mostly commonly caused by *Streptococcus pneumoniae*. In addition, pneumonia patients with prior alcohol use often require more intensive treatment and longer hospital stays due to complications of infection. The initial respiratory tract immune response to *S. pneumoniae* includes the production of pro-inflammatory cytokines and chemokines by resident cells in the upper and lower airways which activate and recruit leukocytes to the site of infection. However, this inflammation must be tightly regulated to avoid accumulation of toxic by-products and subsequent tissue damage. A majority of previous work on alcohol and pneumonia involve animal models utilizing high concentrations of ethanol or chronic exposure and offer conflicting results about how ethanol alters immunity to pathogens. Further, animal models often employ a high bacterial inoculum which may overwhelm the immune system and obscure results, limiting their applicability to the course of human infection. Here, we sought to determine how a more moderate ethanol exposure paradigm affects respiratory function and innate immunity in mice after intranasal infection with 10^4^ colony forming units of *S. pneumoniae.* Ethanol-exposed mice displayed respiratory dysfunction and impaired bacterial clearance after infection compared to their vehicle-exposed counterparts. This altered response was associated with increased gene expression of neutrophil chemokines *Cxcl1* and *Cxcl2* in whole lung homogenates, elevated concentrations of circulating granulocyte-colony stimulating factor (G-CSF), and higher neutrophil numbers in the lung 24 hours after infection. Taken together, these findings suggest that even a more moderate ethanol consumption pattern can dramatically modulate the innate immune response to *S. pneumoniae* after only 3 days of ethanol exposure and provide insight into possible mechanisms related to the compromised respiratory immunity seen in alcohol consumers with pneumonia.

## Introduction

The immune system is influenced by a myriad of environmental factors, including alcohol consumption. In 2019, 69% of U.S. adults aged 26 years or older reported alcohol use in the past month (1). Importantly, social stresses associated with the COVID-19 pandemic have led to increased sales of alcoholic beverages: sales were 3.6% higher in March 2020 and 15.5% higher in April 2021 when compared to a 3-year average from the same month in 2017-2019 (2). Further, alcohol-related deaths have been rising at an astonishingly quick pace in recent years: fatalities due to alcohol rose by 25.9% between 2019 and 2020, compared to a 16.6% increase in age-adjusted mortality rates from all causes (3). Based on this rapidly escalating prevalence of alcohol consumption, we will likely continue to see a rise alcohol-associated morbidity and mortality well into the future.

Alcohol’s effects on the immune system vary based on the amount and duration of consumption. Broadly speaking, alcohol intake can be classified as “moderate” or “excessive” (4). The U.S. Centers for Disease Control and Prevention describe “moderate” alcohol intake as 1-2 drinks per day for males or 1 drink per day for a female (4). “Excessive” alcohol consumption includes binge drinking, or that which brings blood alcohol concentration (BAC) above the “legal limit” for driving (80 mg/dL; usually 5+ drinks for a male or 4+ drinks for a female within 2 hours) and heavy drinking, considered 14+ drinks per week for a male or 7+ drinks for a female (5). In addition, human studies characterize “alcohol use disorder” (AUD) as the inability to modify or discontinue alcohol use despite adverse consequences to one’s personal or work life, and is clinically measured by several social and psychological parameters (6).

Epidemiological studies have shown that moderate alcohol intake is generally associated with protective health effects, such as decreased risk of cardiovascular disease [reviewed in (7)], lower concentrations of circulating inflammatory biomarkers (8), and lower risk of all-cause mortality in humans (9). In contrast, other studies have found an increased risk for all-cause mortality in adults with heavy alcohol consumption [> 14 and > 7 drinks per week for men and women, respectively (9), or 5+ drinks on a single occasion at least once per week in the past year (10)]. Additionally, chronic alcohol users often have health complications associated with alcoholic hepatitis and its treatment, such as invasive aspergillosis (11) and infections of the lower respiratory and urinary tracts (12). Mouse models have similarly shown that excessive ethanol exposure is linked to deleterious effects on immunity; these include decreased responsiveness to Toll-like receptor (TLR) 2, 4, and 9 agonists (13), diminished phagocytic capacity (14, 15), impaired respiratory function (16), and increased airway neutrophils following acute lipopolysaccharide-induced lung injury (17). Neutrophil function is also compromised due to excessive ethanol consumption. For example, chronic ethanol exposure is associated with impaired neutrophil chemotaxis to the airways following pulmonary *Aspergillus fumigatus* infection, along with attenuated phagocytosis, fungal killing, and reactive oxygen species production in *A. fumigatus*-challenged neutrophils from ethanol-fed mice (18). Others have demonstrated that acute ethanol treatment (6 g/kg) decreases neutrophil infiltration into the peritoneal cavity following cecal ligation and puncture, accompanied by diminished production of neutrophil extracellular traps and bacterial killing (19).

Excessive alcohol use has been linked to increased susceptibility to infectious diseases such as pneumonia, with the most common bacterial cause being *Streptococcus pneumoniae* (20). The estimated annual incidence of pneumonia in the United States is 24.8 cases per 10,000 adults (21), and approximately 3.5% of pneumonia patients had an AUD at diagnosis (22). In fact, meta-analyses have found a dose-dependent, linear relationship between alcohol consumption and relative risk for contracting pneumonia (23, 24). In addition, patients with alcohol use disorder are more likely to have severe invasive disease that requires hospitalization (25) and adults who drink excessively (> 60 grams per day) are 4 times more likely to die within 30 days of infection (26).

Innate immune recognition of *S. pneumoniae* by tissue-resident alveolar macrophages or epithelial cells stimulates the production of pro-inflammatory C-X-C motif chemokine ligand 1 (CXCL1) and CXCL2, which recruit and activate cells, such as monocytes, macrophages, and neutrophils to the site of infection (27–29). Additionally, in the context of pulmonary injury, CXCL12 promotes neutrophil migration and retention in the lung (30, 31). Important chemokines for the production and mobilization of granulocytes from the bone marrow are granulocyte colony stimulating factor (G-CSF) and granulocyte macrophage colony stimulating factor (GM-CSF). Following an inflammatory stimuli such as infection, G-CSF and GM-CSF are rapidly produced and secreted by monocytes, macrophages, fibroblasts, and endothelial cells and this corresponds with an increase in neutrophil differentiation and production in the bone marrow (32–34). Indeed, mice lacking G-CSF or its receptor have reduced neutrophil counts in the blood and bronchoalveolar lavage (BAL) fluid following *Pseudomonas aeruginosa* lung infection (35). While the importance of innate immune cells in the response to *S. pneumoniae* has long been appreciated (36–38), excessive accumulation of these cells can be deleterious to host tissue if the inflammatory response is not properly controlled. For example, increased neutrophil recruitment to the lungs following influenza-induced pneumonia in mice is associated with alveolar damage, pulmonary edema, and development of a phenotype similar to that seen in critically ill humans with acute respiratory distress syndrome (39).

Our current knowledge regarding the effects of acute alcohol intoxication on lung immunity is largely based on animal studies using supra-physiologic doses of alcohol (3-5 g/kg) (40, 41), sometimes high enough to raise blood alcohol concentration to 350-550 mg/dL (42, 43). Here, we sought to determine the effect of a 3 day lower-dose alcohol regimen (1.5 g/kg; target BAC of ~80 mg/dL) on the pulmonary response to a clinically relevant intranasal *S. pneumoniae* infection (44).

## Material and Methods

### Mice

Female BALB/cBy mice (Jackson Laboratory) were housed at the University of Colorado Anschutz Medical Campus in specific pathogen-free conditions for at least 2 weeks prior to the start of studies. Animals used in experiments were 3-5 months of age and weighed at least 20 grams. All animal experiments were performed under a protocol approved by the Institutional Animal Care and Use Committee at the University of Colorado Anschutz Medical Campus (protocol number 00087). Animals were housed in a temperature- (72°F ± 2°) and humidity- (35%) controlled room with a 14-hour light cycle (6am-8pm) and 10-hour dark cycle (8pm-6am), and provided with a nestlet for environmental enrichment. Experiments were performed between the hours of 8 and 10 am to minimize confounding effects of circadian variation in corticosterone and other hormones which can influence inflammatory and immune responses (45). For these studies, 3-6 mice comprised each control or treatment group and results are combined from 2-3 individual experiments as indicated in the figure legends.

### Oral Gavage and Measurement of BAC

Mice were orally gavaged with a 20% v/v ethanol solution (1.5 g/kg based on body weight) or vehicle (sterile water) once daily for 3 consecutive days (46). BAC levels were confirmed in each experiment by obtaining blood *via* tail snip at 30 minutes post-gavage and analyzing ethanol levels in a 1:50 dilution of serum using a commercially available kit (BioVision K620). Animals were gavaged between 8 and 9 am to mimic human drinking patterns.

### Bacterial Growth and Infection

Previously frozen glycerol stocks (1 ml) of *Streptococcus pneumoniae* serotype 3 (ATCC 6303) were quickly thawed at 37°C, added to 4 ml of tryptic soy broth (BD 211825), and incubated statically at 37°C/5% CO_2_ until the culture reached mid-log phase (47). Bacteria were washed twice in sterile phosphate buffered saline (PBS), resuspended in an appropriate volume to yield approximately 10^4^ colony forming units (CFU) in 50 µl, and kept on ice until inoculation. One hour after the final gavage, mice were anesthetized with an intraperitoneal injection of 12.5 mg/kg of ketamine and 1.25 mg/kg of xylazine (Webster Veterinary, Sterling, MA), and 50 ul of the prepared inoculum or sterile PBS (Gibco 14190-144) for sham animals was instilled trans-nasally. Mice were held vertically for 1 minute to assist inoculum draining into the lungs. Any mice losing more than 15% of their body weight were humanely euthanized and excluded from analysis. The exact dose of inoculum was quantified in each experiment by serial dilution of the bacterial suspension and plating on tryptic soy agar containing 5% sheep’s blood (Remel R01200).

### Plethysmography

Respiratory function was measured in conscious mice using unrestrained whole-body barometric plethysmography (Buxco Research Systems) as described (16, 48). Briefly, mice were allowed to acclimate in the sealed chamber for 5 minutes and respiratory parameters were measured and recorded for 10 minutes by the manufacturer’s software (Buxco FinePointe). Mean values for each parameter per mouse were used for analysis.

### Lung Bacterial Burden

Whole lungs were removed at 24 hours after infection, placed in 1 ml cold PBS, and homogenized using a Tissue Tearor (Dremel 985370). 100 µl of serially diluted sample was plated on tryptic soy agar containing 5% sheep’s blood (Remel R01200), and CFU were counted after overnight incubation at 37°C/5% CO_2_.

### Lung Macrophage and Neutrophil Quantification by Flow Cytometry

Whole lungs were removed at 24 hours post-infection and dissociated into a single cell suspension per manufacturer’s protocol (Miltenyi 130-095-927). Briefly, separated lung lobes were placed in a C tube (Miltenyi 130-096-334) containing 2.4 ml 1X Buffer S, 100 µl enzyme D, and 15 µl enzyme A. Samples were mechanically disrupted using a GentleMACS instrument (Miltenyi), filtered, and red blood cells were lysed by incubation in Ammonium-Chloride-Potassium (ACK) buffer (Gibco A10492-01) (16). 10^6^ cells per sample were stained in PBS (Gibco) + 1% BSA (Quality Biological Inc K719500ML**)** with the following antibody cocktail: CD45-FITC (clone 30-F11, Biolegend 103108), CD11b-BV650 (clone M1/70, Biolegend 101259), CD11c-BV605 (clone N418, Biolegend 117334), F4/80-PerCP-Cy5.5 (clone BM8, Biolegend 123128), SiglecF-BV421 (clone E50-2440, BD Horizon 562681), and Ly6G-APC-Cy7 (clone 1A8, Biolegend 127624). Samples were resuspended in stabilizing fixative (BD 338036), run on an LSRII flow cytometer (BD Biosciences), and data were analyzed using FlowJo software v10.7.1 (BD Life Sciences).

### Quantitative Real-Time PCR

RNA was extracted from homogenized lung tissue using the RNeasy Mini kit (Qiagen 74106) and reverse transcribed to cDNA (BioRad 1708891) following the manufacturers’ protocols (49). Equal quantities of cDNA were added to a Taqman master mix (Life Technologies 4304437) containing either *Cxcl1* (ThermoFisher Mm04207460_m1), *Cxcl2* (ThermoFisher Mm00436450_m1), *Cxcl12* (ThermoFisher Mm00445553_m1), *Ly6g* (ThermoFisher Mm04934123_m1), *Csf2* (ThermoFisher Mm01290062_m1) or *Csf3* (Mm00438334_m1), and *Gapdh* as endogenous control (Thermo Fisher 4352339E). Real-time quantitative PCR was performed using the QuantStudio 3 Real-Time PCR System (ThermoFisher Scientific) and analyzed using the ΔΔCt algorithm (50).

### Enzyme-Linked Immunosorbent Assay (ELISA)

Blood was collected from mice *via* cardiac puncture immediately following euthanasia and allowed to coagulate for 30 minutes before serum separation by centrifugation. Granulocyte-colony stimulating factor (G-CSF) was measured in serum samples according to manufacturer’s protocol (R&D Systems DY414).

### Lung Histology and Immunohistochemistry (IHC)

The left lung lobe was inflated with 10% formalin (Fisher SF98-4), fixed overnight, and kept in 70% ethanol until processing. Paraffin-embedded tissue was sectioned (5 µm) and stained using hematoxylin and eosin (H&E) or IHC antibodies and scored in a blinded fashion by an experimental pathologist. IHC was performed using primary antibodies against Ly6G (1:250, BD Biosciences 551459) and *S. pneumoniae* (1:1000; Novus Biologicals NB100-64502), and detected using ImmPress polymer reagents (Vector Laboratories). Antigen expression was visualized with 3,3’Diaminobenzidine and alkaline phosphatase according to manufacturer’s protocol (Vector Laboratories). Sections were counterstained with Hematoxylin QS and slides were coverslipped using VectaMount medium (Vector Laboratories).

### Quantification of H&E- and IHC-Stained Lung Sections

Semi-quantitative assessment of the following injury criteria in the H&E-stained sections was performed: gross accumulations of inflammatory cells in the lung parenchyma (0-6), presence of leukocytes in the airways (0-2), peri-vascular inflammatory cell accumulation (0-2) or edema (0-2), and proteinaceous material in the alveolar space (0-2). Analysis of IHC staining intensity was achieved by capturing histologic images [enough 40x images per animal to completely cover the whole IHC-stained lung cross-section (5-8 per animal)] on an Olympus BX51 (Waltham, MA) microscope equipped with a 4 megapixel Macrofire digital camera using the PictureFrame Application 2.3 (Optronics, Goleta, CA). Images were then imported into Slidebook (3I, Denver, CO) for quantification. Data are expressed as percent IHC positive pixels (51). Phagocytosis of *S. pneumoniae* was measured by capturing stitched images with a light microscope equipped with a motorized XY-stage at 400x magnification (Olympus IX83) and CellSens software (version 1.16, Olympus Life Sciences). Manual quantification of 8-9 non-overlapping 400x images per animal was performed and phagocytosis was calculated as the number of cells with internalized *S. pneumoniae* relative to total nucleated cells. Average percentage phagocytosis per group is presented.

### Statistical Analysis

Data were analyzed with Graph Pad Prism software for Windows version 9.2.0 (GraphPad Software, San Diego, California USA) using an unpaired two-tailed t test with Welch’s correction or one-way ANOVA as appropriate and indicated in figure legends. p value < 0.05 was considered to represent a significant difference between treatment groups.

## Results

### Ethanol-Exposed Mice Have Increased Lung Bacterial Burden After *S. pneumoniae* Infection Compared to Vehicle-Exposed Animals

**Figure 1:**
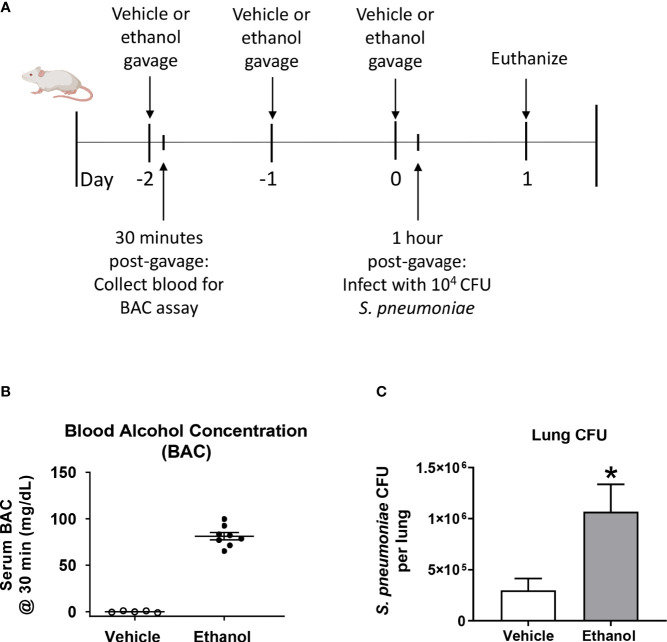
Effect of ethanol exposure on blood alcohol concentration (BAC) and *S. pneumoniae* burden in the lung. **(A)** Schematic diagram of ethanol exposure and *S. pneumoniae* infection. **(B)** Representative BAC measurements from serum at 30 minutes post-gavage. Each dot represents the value from a single animal from one experiment. The line for each group represents average value ± SEM. **(C)** Whole lungs were collected at 24 hours post-infection, homogenized, and plated on agar. *S. pneumoniae* colony forming units (CFU) were enumerated and data are presented as mean ± SEM. n = 4-6 mice per group per experiment and data are combined from 3 individual experiments. *p < 0.05 by unpaired t test.

To test our hypothesis that ethanol exposure impairs clearance of *S. pneumoniae* from the lungs, we gavaged mice with vehicle (water) or ethanol (1.5 g/kg) and intra-nasally instilled *S. pneumoniae* or PBS (**Figure 1A**). This dose of ethanol raised serum BAC to approximately 80 mg/dL at 30 minutes post-gavage (**Figure 1B**). Our results indicate that ethanol-exposed infected mice had significantly higher average *S. pneumoniae* CFU in whole lung homogenates at 24 hours post-infection (**Figure 1C**). Importantly, since we are introducing 10^4^ CFU *S. pneumoniae via* an intra-nasal route (vs. direct administration of bacteria to the lungs *via* an intra-tracheal infection), our results confirm that the bacteria are able to withstand the initial immune response in the upper respiratory tract to reach the lungs, establish an infection, and begin to replicate.

### Ethanol-Exposed Mice Have Impaired Respiratory Function After *S. pneumoniae* Infection Compared to Vehicle-Exposed Animals

**Figure 2:**
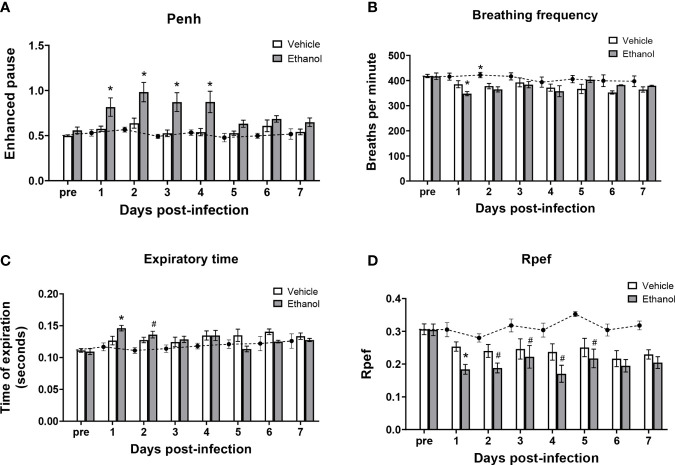
Effect of ethanol consumption on respiratory function following infection. Enhanced pause (penh) **(A)**, breathing frequency **(B)**, time of respiratory expiration (Te) **(C)**, and Rpef (time to peak expiratory flow as a fraction of Te) **(D)** were measured by unrestrained whole-body plethysmography before infection and daily for 7 days post-infection. Black circles with dashed line represents values from vehicle-treated uninfected mice; solid bars represent values from infected animals (except for the “pre” values, which were obtained in vehicle- and ethanol-exposed mice prior to infection). Data are presented as mean ± SEM. n = 3-6 mice per group per experiment and data are combined from 3 individual experiments. *p < 0.05 compared to vehicle-exposed infected animals, #p < 0.05 compared to uninfected animals by one-way ANOVA.

To determine the effect of ethanol consumption on respiratory function in our infected animals, we performed unrestrained whole-body plethysmography prior to infection and up to 7 days after infection. Here, we report respiratory rate [breaths per minute (bpm)], enhanced pause ratio (penh) – a dimensionless index of airflow patterns as a mouse breathes (43), expiration time (Te), and Rpef – the time required to reach peak expiratory flow relative to Te (44). Before infection, all parameters were similar between our vehicle- and ethanol-exposed groups; therefore, we used vehicle-exposed uninfected animals as our control group throughout the 7-day time course (**Figure 2A–D**). When comparing respiratory parameters between infected groups, we noted a significantly higher penh value in our ethanol- compared to vehicle-exposed infected mice from days 1-4 post-infection (**Figure 2A**), decreased breathing frequency in the ethanol-exposed infected animals at 24 hours (**Figure 2B**), increased expiratory time in ethanol-exposed animals at 24 hours (**Figure 2C**), and a lower Rpef value in ethanol- compared to vehicle-exposed infected mice at 24 hours (**Figure 2D**). Notably, penh values in the vehicle-exposed infected mice did not differ from the uninfected mice through 7 days of infection (**Figure 2A**).

### Ethanol-Exposed Mice Have Increased Numbers of Neutrophils, but Similar Numbers of Macrophages, in the Lung 24 Hours After Infection Compared to Vehicle-Exposed Mice

**Figure 3:**
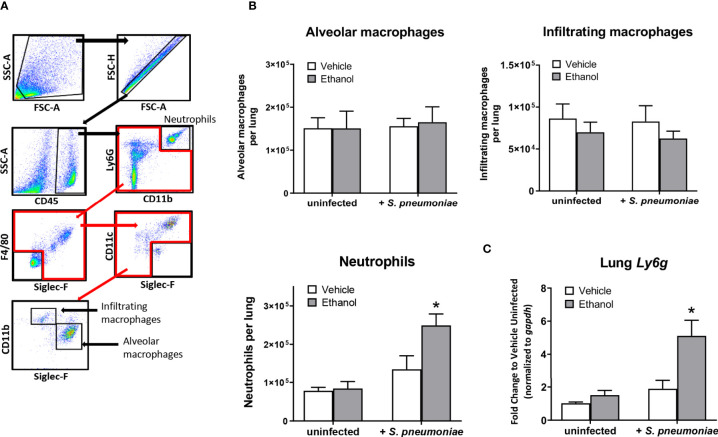
Effect of ethanol consumption on macrophage and neutrophil numbers in the lung following infection. **(A)** Flow cytometry gating strategy to identify neutrophils, alveolar macrophages, and infiltrating macrophages in lung homogenates. **(B)** Average number of pulmonary cell subsets in lung homogenate at 24 hours post-infection. **(C)** RNA from lung homogenates was isolated at 24 hours post-infection and cDNA was analyzed by quantitative PCR for expression of *Ly6g*; target gene expression is normalized to *gapd*h and presented as fold change to vehicle-treated uninfected mice. Data are presented as mean ± SEM. n = 3-6 mice per group per experiment and data are combined from 2 individual experiments. *p < 0.05 compared to all other groups by one-way ANOVA.

Since our initial results showed that ethanol administration is associated with increased pulmonary bacterial burden and impaired respiratory function at 24 hours, we wondered if this was due to ineffective trafficking of immune cells to the infection site at this time point. To test this, we used flow cytometry to quantify neutrophils and macrophages, important early responders to *S. pneumoniae* infection, in single cell suspensions of whole lung homogenates. We identified neutrophils as CD45^+^CD11b^+^Ly6G^hi^, and distinguished tissue-resident alveolar macrophages from infiltrating macrophages based on SiglecF and CD11b expression (**Figure 3A**). Alveolar macrophages are designated as CD45^+^F4/80^+^SiglecF^+^CD11b^neg/dim^, while infiltrating macrophages are CD45^+^F4/80^+^SiglecF^neg/dim^CD11b^+^ (45). The number of cells in these subsets did not differ in our uninfected mice based on vehicle or ethanol treatment (**Figure 3B**). However, we found increased numbers of neutrophils in both infected groups relative to uninfected animals at 24 hours (**Figure 3B**), indicative of an innate immune response to the infection. When comparing vehicle-exposed infected animals to their ethanol-exposed counterparts, we found that ethanol exposure resulted in 1.8-fold more neutrophils in the lungs of infected animals at 24 hours but noted no difference in the number of alveolar or infiltrating macrophages (**Figure 3B**). Finally, to complement our result of increased neutrophils in the lungs of ethanol-exposed animals after infection, we performed quantitative PCR on whole lung homogenates for *Ly6g* expression. We observed 5.0- and 2.7-fold higher *Ly6g* transcript in lung homogenates of ethanol-exposed infected animals compared to uninfected animals or vehicle-exposed infected animals, respectively (**Figure 3C**).

### Ethanol Exposure Prior to *S. pneumoniae* Infection Leads to Increased Pulmonary Expression of Pro-Inflammatory Cytokines and Chemokines, and Increased G-CSF in the Serum

**Figure 4:**
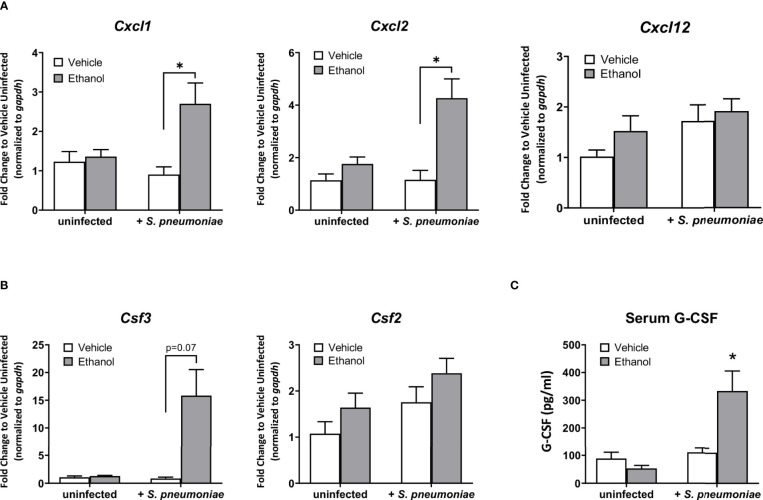
Effect of ethanol exposure on chemokine gene expression in the lung and serum granulocyte-colony stimulating factor (G-CSF) levels after infection. **(A, B)** Gene expression as indicated in graph titles was measured as described in **Figure 3**. Target gene expression is normalized to *gapd*h and presented as fold change to vehicle-treated uninfected mice. **(C)** Serum concentration of G-CSF at 24 hours post-infection as measured by ELISA. Data are presented as mean ± SEM. n = 3-5 per group per experiment and represent averages from 3 individual experiments. *p < 0.05 by one-way ANOVA.

Based on our results showing an increased number of lung neutrophils in ethanol-exposed infected animals observed by flow cytometry, we evaluated pulmonary expression of the chemokine genes *Cxcl1*, *Cxcl2*, and *Cxcl12*, important neutrophil chemoattractants and activators produced during the inflammatory response to infection (26, 46). Additionally, we measured pulmonary expression of *Csf2* and *Csf3*, the genes encoding for granulocyte macrophage-colony stimulating factor (GM-CSF) and granulocyte colony-stimulating factor (G-CSF), respectively, important cytokines for inducing granulopoiesis and the release of granulocytes from the bone marrow (47), along with serum levels of G-CSF. Our data show that, compared to vehicle-exposed infected animals, the ethanol-exposed infected mice had significantly higher expression of *Cxcl1* (3.0-fold) and *Cxcl2* (3.7-fold), and no difference in *Cxcl12*, in lung homogenates at 24 hours (**Figure 4A**). Further, we found no difference in *Csf2* expression, but rather elevated levels of *Csf3* transcript in the lung and G-CSF in the serum (17.2-fold and 3.0-fold higher, respectively), in ethanol- compared to vehicle-exposed infected animals (**Figures 4B, C**). We noted no difference in pulmonary gene expression or serum G-CSF levels between our vehicle- or ethanol-exposed uninfected groups (**Figure 4**).

### Ethanol Exposure Alters Leukocyte and *S. pneumoniae* Localization in the Lung at 24 Hours Following Infection

**Figure 5:**
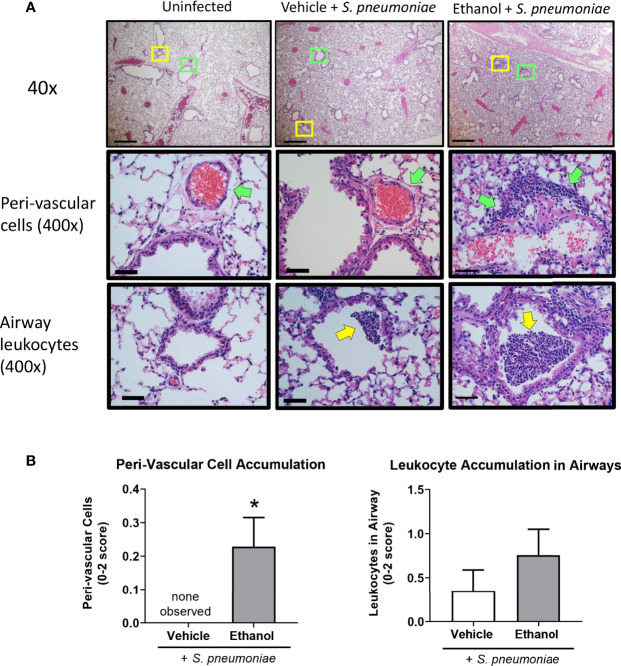
Effect of ethanol exposure on lung inflammation following *S. pneumoniae* infection. **(A)** Representative images of lungs from uninfected and infected mice at 24 hours. Top panel = 40x magnification, scale bar = 500 µm. Boxes denote the magnified areas in the middle and bottom panels. Middle and bottom panels = 400x magnification, scale bar = 50 µm; green arrows denote peri-vascular cells and yellow arrows denote airway leukocytes. **(B)** Quantitative score for criterion of peri-vascular and airway cell accumulation (0-2 score). Data are presented as mean ± SEM. n = 3-6 mice per group per experiment and data are combined from 2 individual experiments. *p < 0.05 by unpaired t test.

Due to the increased number of neutrophils in the lungs of ethanol-exposed infected animals detected by flow cytometry at 24 hours, we analyzed histological markers of inflammation and injury in H&E-stained lung sections. We found that ethanol-exposed infected animals had significantly increased peri-vascular cell accumulation at 24 hours and a trend toward more leukocytes in the lumen of the larger airways compared to vehicle-exposed infected animals (**Figures 5A, B**). We failed to observe a difference in the other individual scoring criteria—gross accumulation of inflammatory cells in the lung parenchyma, peri-vascular edema, or proteinaceous material in the alveolar space (data not shown).

**Figure 6:**
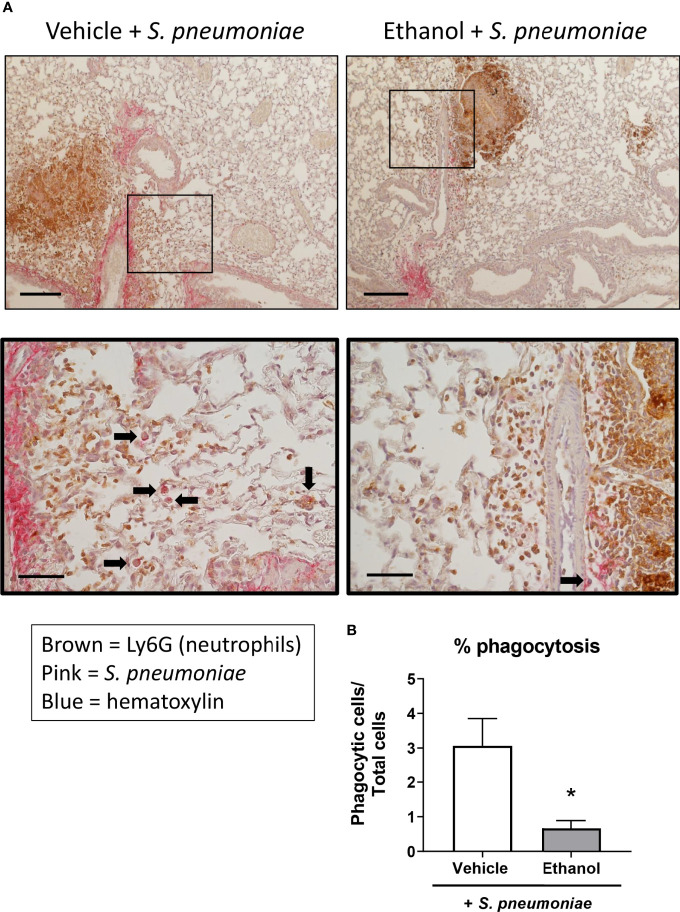
Effect of ethanol exposure on neutrophil and S. pneumoniae localization and bacterial phagocytosis after infection. **(A)** IHC staining was performed onformalin-fixed lung sections with antibodies against Ly6G for neutrophils (brown) and *S. pneumoniae* (pink) and counterstained with hematoxylin. Representative images are at 100x magnification for the top panel (scale bar = 200μm) and 400x magnification for the bottom panel (scale bar = 50 μm). **(B)** Percent phagocytosis of *S. pneumoniae* as measured by the ratio of cells with internalized bacteria to total nucleated cells. Black arrows denote cells with internalized S. pneumoniae. Data are presented as mean ± SEM. n = 3-4 mice per group per experiment and are representative of 2 individual experiments. *p < 0.05 by unpaired t test.

Next, since we noted altered leukocyte localization in the lungs by H&E and a difference in neutrophil numbers and transcript by flow cytometry and qPCR, respectively, we evaluated neutrophil and bacterial localization after infection utilizing IHC staining. Quantification of staining (as assessed by percent positive pixels) showed a great deal of variation between animals within groups, and thus, no statistical difference was observed in the levels of Ly6G or *S. pneumoniae* antigen in lung sections from our vehicle- and ethanol-exposed infected animals (**Supplementary Figure 1**). We noted an increased presence of neutrophils in the infected groups compared to vehicle-exposed uninfected animals (**Supplementary Figure 1**), with neutrophil localization near *S. pneumoniae* in both infected groups (**Figure 6**). Additionally, we observed a 4.6-fold higher percentage of internalized *S. pneumoniae* in macrophages of vehicle- compared to ethanol-exposed infected animals (**Figure 6**).

## Discussion

Excessive alcohol consumption weakens the ability of the immune system to effectively respond to pathogens (52). Here, we describe those 3 consecutive days of a more moderate ethanol exposure than what is typically used in the literature (**Figures 1A, B**) can significantly alter the early pulmonary response to *Streptococcus pneumoniae*. Although the peak BAC in our studies is approximately 80 mg/dL, considered the “legal limit” for driving in humans, it is likely that the BAC levels for intoxication in mice are comparably lower due to increased metabolism of ethanol in rodents compared to humans (53). Nonetheless, we found that ethanol exposure increases bacterial burden in the lung and decreases respiratory function within 24 hours after *S. pneumoniae* infection (**Figures 1C**, **2**). Our results showing increased bacterial burden in the lungs of ethanol-exposed mice (**Figure 1C**) is in line with previous studies utilizing pathogens such as *S. pneumoniae* (54), *Klebsiella pneumoniae* (55, 56), and *Escherichia coli* (57). However, the animal models in these studies use supra-physiological levels of acute ethanol (often enough to raise BAC well above 350 mg/dL) or binge-on-chronic ethanol feeding [Lieber-DeCarli diet (58) for a total of 10 days with a 4 g/kg ethanol gavage on days 5 and 10 (55, 56)] before infection, making it challenging to directly compare results. Even so, our work expands upon previous findings by showing a similar response of increased bacterial burden in animals exposed to a much lower and shorter three-day ethanol exposure regimen (1.5 g/kg) and infected with a lower *S. pneumoniae* inoculum (10^4^ CFU). We believe that our model better recapitulates how humans who drink alcohol may acquire bacterial pneumonia *via* respiratory droplets, as our mice are given an oral gavage of ethanol at a more moderate level and then infected intranasally. Furthermore, humans tend to drink alcohol for social motives (59) putting them in close proximity to others who may be infected, or more commonly, those who are asymptomatic carriers of *S. pneumoniae* (60).

Previous studies have found that respiratory disease is associated with impaired lung function using whole body plethysmography, including pneumococcal (61) and SARS-CoV2 infection (62), and bleomycin-induced lung injury (63). Further, our group has shown that multi-day ethanol exposure leads to respiratory dysfunction following traumatic injury, such as a cutaneous scald (16, 64). The increased penh values suggest altered airflow patterns in response to airway inflammation (65, 66), while the higher expiratory time and corresponding decreased Rpef values suggest airway narrowing/obstruction (67, 68) and airway collapse with increased airflow resistance (69, 70) in ethanol- compared to vehicle-exposed infected mice. Increased leukocyte accumulation, pulmonary edema, and alveolar wall thickening observed histologically (**Figure 5A**; **Supplementary Figure 2**) likely contribute to the impaired respiratory function observed by plethysmography. One caveat to our lung function data is that we observed mortality during the studies so respiratory parameters are representative of surviving animals only. On day 4, 6% and 14% of the vehicle- and ethanol-exposed infected animals, respectively, died. An additional 12% and 8%, respectively, of the remaining animals died on day 5; we did not observe any further mortality through day 7 post-infection (data not shown). Nevertheless, our study is the first to our knowledge that demonstrates an effect of multi-day lower-dose ethanol exposure on respiratory parameters following *S. pneumoniae* infection.

Neutrophils are critical early responders to respiratory pathogens, but their presence and activity at infection sites can be detrimental if not properly controlled. Using several different pathogens, animal studies have demonstrated that the host is more susceptible to severe pneumonia and death when neutrophils are depleted or otherwise unable to reach the lung (36, 71), but also when excessive neutrophil accumulation and their ineffective clearance leads to tissue damage (72–75). Our results show that ethanol-exposed mice had a higher pulmonary bacterial burden at 24 hours after infection (**Figure 1C**) despite increased numbers of pulmonary neutrophils (**Figure 3B**) and correspondingly higher expression of chemokines involved in neutrophil recruitment, such as *Cxcl1* and *Cxcl2* (**Figure 4A**). Likewise, the upregulation of *Csf3* in the lungs and increased levels of G-CSF in the serum of ethanol-exposed infected mice (**Figures 4B, C**) further suggests an immune response geared toward granulopoiesis and neutrophil homing to the lung. Indeed, others have shown that G-CSF mRNA levels are markedly increased in lung tissue from animals challenged intratracheally with *E. coli* but did not observe appreciable levels of G-CSF mRNA in spleen, liver, or kidney tissue from the same animals (76). This suggests that the lung is a primary source of G-CSF production early after pulmonary infection.

Previous work from our lab has shown that prior ethanol exposure leads to excessive accumulation of neutrophils (77, 78) and apoptotic cells (78) in the lungs of mice up to 24 hours following burn injury, compared to vehicle-treated injured mice. Although not directly tested in these studies, others have shown that ethanol exposure decreases phagocytic capacity in both alveolar macrophages (14, 15, 79–81) and neutrophils (82–84), along with decreased efferocytosis by alveolar macrophages (17). Our results showing increased bacterial burden in the lung despite higher numbers of neutrophils may suggest that either the tissue-resident alveolar macrophages or the infiltrating neutrophils are less efficient at properly phagocytosing and/or breaking down *S. pneumoniae* within the first 24 hours after infection. Our IHC staining of infected lung tissue would suggest the former (**Figure 6**), although we cannot rule out that the recruited neutrophils are also functionally impaired. Additionally, the presence of Ly6G-negative airway cells could indicate apoptotic macrophages that were not detected by flow cytometry. It is also possible that ethanol treatment in our mice indirectly alters neutrophil apoptosis due to the hyper-inflammatory lung microenvironment following infection (85) and/or impairs efferocytosis of apoptotic neutrophils by alveolar macrophages or infiltrating macrophages; this question merits further evaluation and clarification. Indeed, others have reported delayed neutrophil apoptosis after acute ethanol exposure followed by a “second hit” to the immune system (86, 87). It has been shown that blood neutrophils from ethanol-exposed burned animals had decreased expression of pro-apoptotic proteins such as caspase-3 and Bax, and correspondingly decreased apoptosis as measured by histone-associated DNA fragments (86). Further, delayed neutrophil death appears to be a common characteristic of human inflammatory lung diseases such as cystic fibrosis, pneumonia, and idiopathic fibrosis, as well as in cancer with associated neutrophilia (87). Importantly, in our model, if recruited pulmonary neutrophils are not able to properly die *via* apoptosis and be cleared by alveolar macrophages, they may undergo secondary necrosis, releasing toxic mediators such as elastase and reactive oxygen species, and inducing lung damage in our ethanol-exposed animals (88, 89).

Inflammation is a necessary process to restore homeostasis after infection. Resident immune cells in the upper and lower respiratory tract detect pathogen associated molecular patterns on invading microorganisms and initiate a signaling cascade leading to mobilization and migration of leukocytes to the site of infection. The lungs of our infected animals showed an early accumulation of peri-vascular and airway leukocytes in animals with prior ethanol exposure (**Figures 4A, B**). This suggests that recruited immune cells are able to reach the lungs but appear to be less efficient at clearance of *S. pneumoniae* as noted by CFU counts at 24 hours post-infection in our ethanol-exposed mice (**Figure 1C**). While we failed to see differences in the number of alveolar or infiltrating macrophages in lung homogenates by flow cytometry (**Figure 3**), it is possible that ethanol exposure impairs monocyte trafficking to the lung, as others have previously shown (90).

Immunohistochemistry staining of lung sections provided valuable insight into the effect of ethanol on neutrophil and bacterial antigen localization, as well as bacterial internalization, following *S. pneumoniae* infection. We did not observe any gross difference in neutrophil proximity to *S. pneumoniae*, suggesting that neutrophils are able to migrate toward the infection site at 24 hours post-infection in our model. However, it is possible that neutrophil chemotaxis is impaired at earlier or later time points post-infection, or in other areas of the lung. Further, there was no significant difference in neutrophil or bacterial antigen staining intensity (**Supplementary Figure 1**), however, we are careful not to rely solely on this result since the quantification is from a single tissue section from each animal. Additionally, the *S. pneumoniae* antibody used for IHC is a whole cell serotype blend and will therefore bind to viable and non-viable bacteria and also closely related bacterial species; therefore, quantification of *S. pneumoniae* antigen by IHC may not directly relate to CFU counts of viable bacteria from lung homogenates.

The results presented here require further clarification in future studies. A strength of this work is the demonstration of increased neutrophils and *S. pneumoniae* in whole lung homogenates of ethanol-exposed infected animals at 24 hours, and visualization of the localization of each within the lung. In future experiments, we will characterize the number and functional capacity of neutrophil and macrophage populations in the airways following infection by isolation of these cells from BAL fluid (91). Advanced flow cytometry analysis of blood, BAL fluid, and whole lung homogenates will identify the changing inflammatory cell populations and discriminate cell death (apoptosis or necrosis) in each. Additionally, it would be useful to determine which subset of pulmonary cells are expressing *Cxcl1*, *Cxcl2* and *Csf3*, as this may yield a focused therapeutic target to improve health outcomes in alcohol consumers with pneumococcal pneumonia. Finally, because *S. pneumoniae* was still present in the lungs at 24 hours post-infection, it is imperative to examine the later innate and subsequent adaptive immune response in our model and determine whether complete resolution of the infection is altered due to ethanol exposure.

In summary, we show here that ethanol exposure—at a dose relevant to human consumption—results in higher lung bacterial burden despite an increased presence of pulmonary neutrophils following intranasal *S. pneumoniae* infection. An accumulation of leukocytes was visualized in the airways and peri-vascular space, and likely contributes to the respiratory dysfunction seen in our infected animals. Additionally, we noted differences in *S. pneumoniae* internalization in macrophages from our ethanol-exposed infected animals, which likely contributes to the increased inflammation noted in our mice and could lead to delayed resolution of the infection. Taken together, these findings contribute to our knowledge of how short-term ethanol consumption at physiological doses can alter pulmonary immunity to respiratory infection. Future studies aimed at understanding the mechanisms underlying this exacerbated neutrophilic response could improve health outcomes in pneumonia patients who drink alcohol.

## Data Availability Statement

The raw data supporting the conclusions of this article will be made available by the authors, without undue reservation.

## Ethics Statement

The animal study was reviewed and approved by IACUC University of Colorado Anschutz Medical Campus.

## Author Contributions

HH and KN designed and carried out the experiments. HH analyzed the data and wrote the manuscript. RM provided guidance on study design, and analyzing and interpreting flow cytometry data. DB provided critical input on experimental strategy and panel design for the flow cytometry experiments. DO blindly scored the H&E sections and provided interpretation of the lung histology. EK supervised the project. All authors provided feedback on results and critically reviewed the manuscript. All authors contributed to the article and approved the submitted version.

## Funding

This work was supported in part by NIH F31 AA027687 (HH), R21 AA026295 (EK), R01 AG018859 (EK), R35 GM131831 (EK), and VA 1 I01 BX004335 (EK).

## Conflict of Interest

The authors declare that the research was conducted in the absence of any commercial or financial relationships that could be construed as a potential conflict of interest.

## Publisher’s Note

All claims expressed in this article are solely those of the authors and do not necessarily represent those of their affiliated organizations, or those of the publisher, the editors and the reviewers. Any product that may be evaluated in this article, or claim that may be made by its manufacturer, is not guaranteed or endorsed by the publisher.

## Supplementary Material

Supplementary Figure 1 Effect of ethanol exposure on Ly6G and *S. pneumoniae* antigen levels. IHC staining was performed on formalin-fixed lung sections with antibodies against Ly6G for neutrophils (brown) and *S. pneumoniae* (pink) and counterstained with hematoxylin. **(A)** Representative images of lung sections from vehicle-exposed uninfected animals; scale bars = 500 µm for 40x magnification and 50 µm for 400x magnification. **(B)** Quantification of staining intensity as measured by percent positive pixels per lung. Images are representative of 3-6 mice per group per experiment from 2 individual experiments.

Supplementary Figure 2 Effect of ethanol exposure on alveolar wall thickening and edema. Representative images at 200x of lungs from uninfected and infected mice at 24 hours. Scale bar = 100 µm; black arrows denote areas of alveolar wall thickening and edema, green arrows denote cellular accumulation and airway obstruction. Images are representative of 3-6 mice per group per experiment from 2 individual experiments.
