# Supplementary figures and images for: Ethanol Intoxication Impairs Respiratory Function and Bacterial Clearance and Is Associated With Neutrophil Accumulation in the Lung After Streptococcus pneumoniae Infection
Source: Front Immunol. 2022 May 4;13:884719. doi: 10.3389/fimmu.2022.884719 (PMC9116899; doi:10.3389/fimmu.2022.884719)

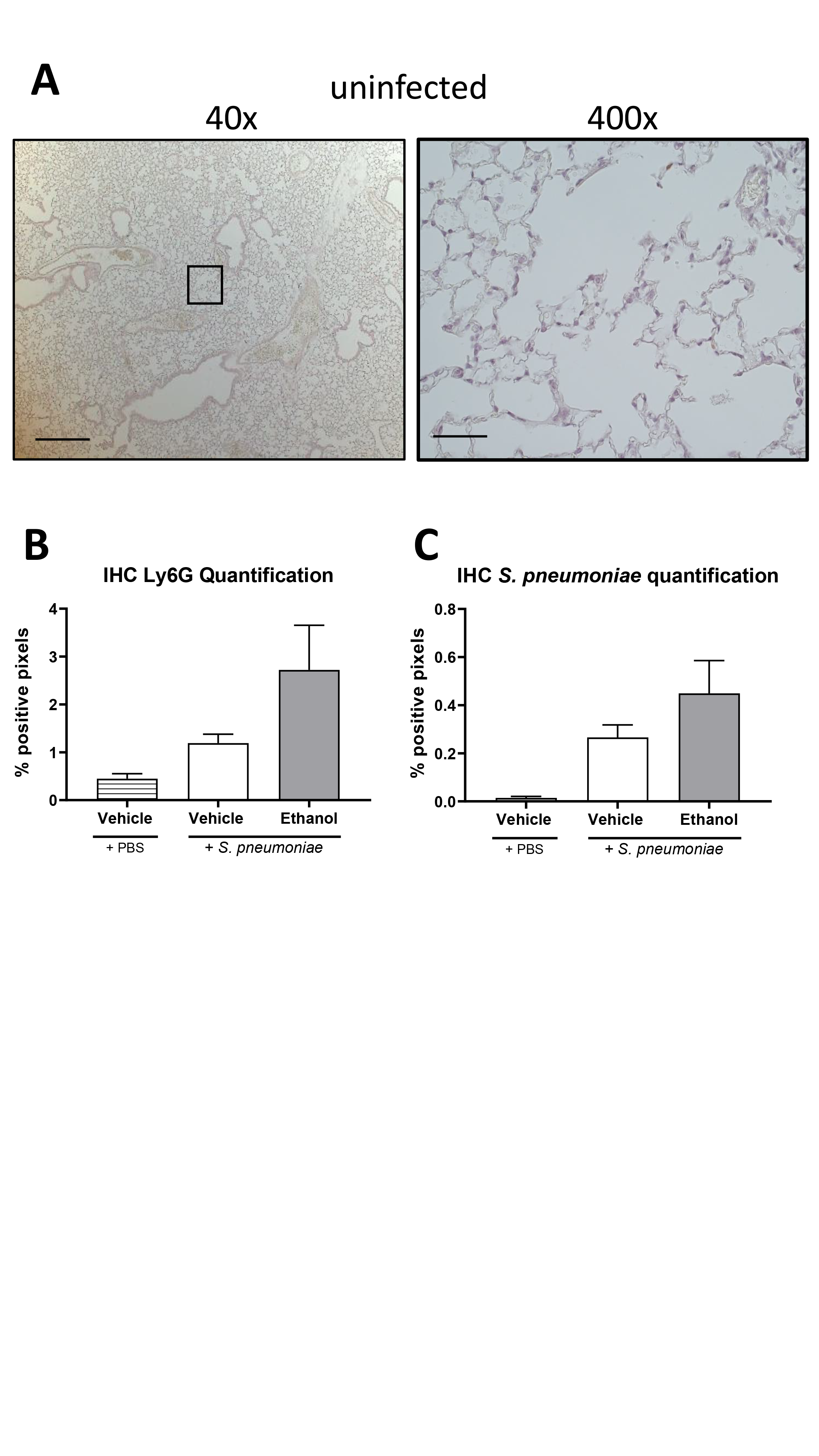

Supplement: Supplementary Figure 1 — Effect of ethanol exposure on Ly6G and S. pneumoniae antigen levels. IHC staining was performed on formalin-fixed lung sections with antibodies against Ly6G for neutrophils (brown) and S. pneumoniae (pink) and counterstained with hematoxylin. (A) Representative images of lung sections from vehicle-exposed uninfected animals; scale bars = 500 µm for 40x magnification and 50 µm for 400x magnification. (B) Quantification of staining intensity as measured by percent positive pixels per lung. Images are representative of 3-6 mice per group per experiment from 2 individual experiments. [file Image_1.tif]

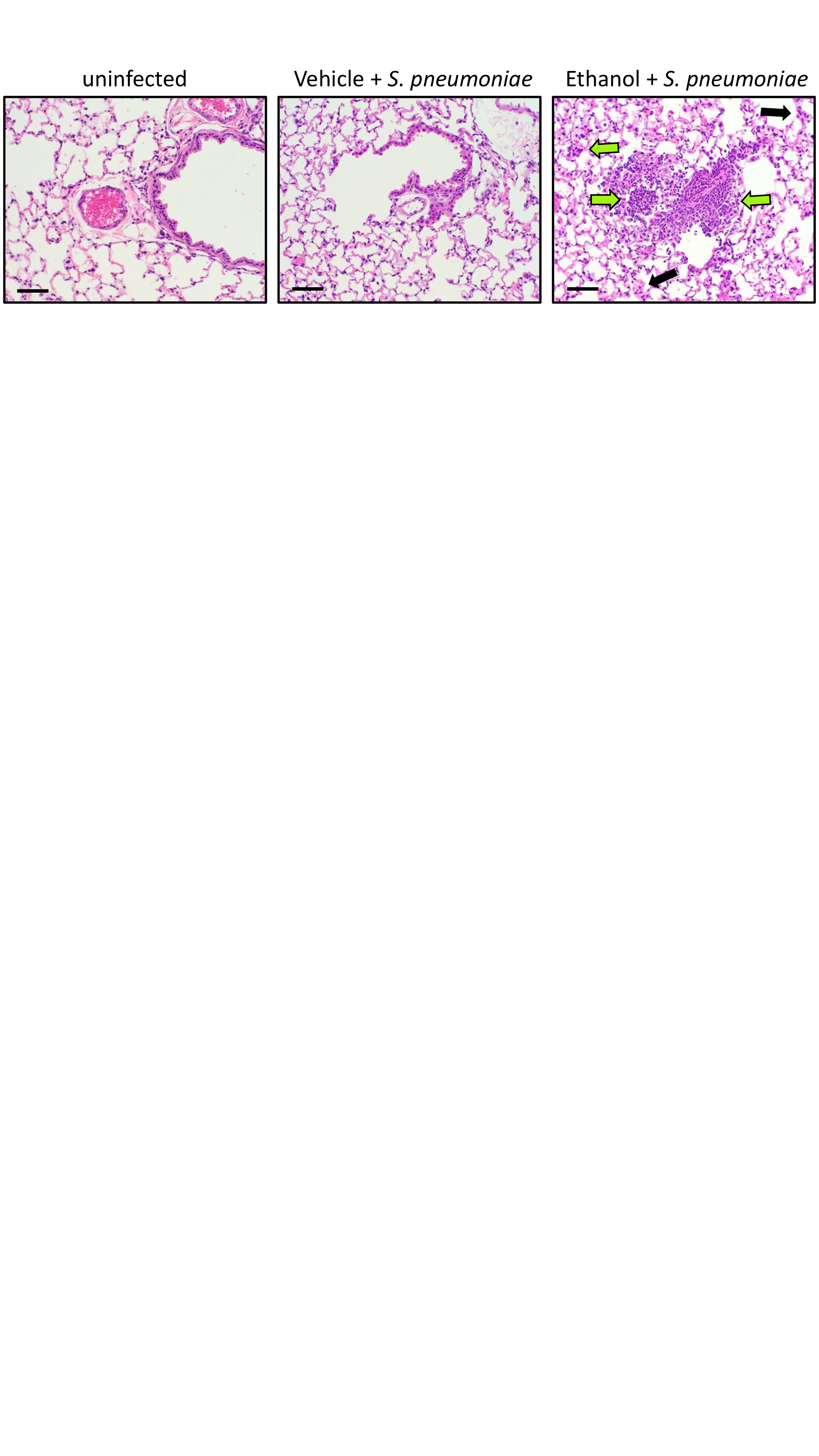

Supplement: Supplementary Figure 2 — Effect of ethanol exposure on alveolar wall thickening and edema. Representative images at 200x of lungs from uninfected and infected mice at 24 hours. Scale bar = 100 µm; black arrows denote areas of alveolar wall thickening and edema, green arrows denote cellular accumulation and airway obstruction. Images are representative of 3-6 mice per group per experiment from 2 individual experiments. [file Image_2.tif]
